# Epirubicin and Non-Muscle Invasive Bladder Cancer Treatment: A Systematic Review

**DOI:** 10.3390/jcm13133789

**Published:** 2024-06-27

**Authors:** Sever Chiujdea, Matteo Ferro, Mihai Dorin Vartolomei, Giuseppe Lucarelli, Kensuke Bekku, Akihiro Matsukawa, Mehdi Kardoust Parizi, Jakob Klemm, Ichiro Tsuboi, Tamas Fazekas, Stefano Mancon, Shahrokh F. Shariat

**Affiliations:** 1 Doctoral School, George Emil Palade University of Medicine, Pharmacy, Sciences and Technology, 540142 Târgu Mureș, Romania; severchiujdea@gmail.com (S.C.); matteo.ferro@ieo.it (M.F.); 2 Urology Department, European Institute of Oncology, 20122 Milan, Italy; 3 Department of Urology, Comprehensive Cancer Center, Medical University of Vienna, 1090 Vienna, Austria; shahrokh.shariat@meduniwien.ac.at; 4 Department of Precision and Regenerative Medicine and Ionian Area Urology, Andrology and Kidney Transplantation Unit, 70124 Bari, Italy; giuseppe.lucarelli@uniba.it; 5 Department of Urology, Okayama University Graduate School of Medicine, Dentistry and Pharmaceutical Sciences, Okayama 700-8558, Japan; gmd421030@s.okayama-u.ac.jp; 6 Department of Urology, Jikei University School of Medicine, Tokyo 143-8541, Japan; a.matsu.audi1055g@gmail.com; 7 Department of Urology, Shariati Hospital, Tehran University of Medical Sciences, Tehran 14878-92855, Iran; m.kardoust@yahoo.com; 8 Department of Urology, University Medical Center Hamburg-Eppendorf, 20359 Hamburg, Germany; jakob.klemm@meduniwien.ac.at; 9 Department of Urology, Shimane University Faculty of Medicine, Shimane 693-8501, Japan; ichiro.tsuboi0810@gmail.com; 10 Department of Urology, Semmelweis University, 1085 Budapest, Hungary; fazekastamas192@gmail.com; 11 Department of Biomedical Sciences, Humanitas University, 20090 Pieve Emanuele, Italy; manconstefano@gmail.com; 12 Department of Special Surgery, Division of Urology, Jordan University Hospital, The University of Jordan, Amman 11942, Jordan; 13 Karl Landsteiner Society, Karl Landsteiner Institute of Urology and Andrology, 1090 Vienna, Austria; 14 Department of Urology, Weill Cornell Medical College, New York, NY 10065, USA; 15 Department of Urology, University of Texas Southwestern, Dallas, TX 75390, USA; 16 Research Center for Evidence Medicine, Urology Department Tabriz University of Medical Sciences, Tabriz 51656-65811, Iran

**Keywords:** epirubicin, intravesical instillations, bladder cancer, chemotherapy, adjuvant treatment

## Abstract

**(1) Background:** Intravesical chemotherapy is the standard of care in intermediate-risk non-muscleinvasive bladder cancer (NMIBC). Different agents are used across the world based on availability, cost, and practice patterns. Epirubicin (EPI), one of these agents, has been used by many centers over many decades. However, its true differential efficacy compared to other agents and its tolerability are still poorly reported. We aimed to assess the differential efficacy and safety of intravesical EPI in NMIBC patients. **(2) Methods**: This study aimed to systematically review the efficacy and safety profile of Epirubicin (EPI) in the management of non-muscle invasive bladder cancer (NMIBC) compared to other adjuvant therapies. A systematic search of the PUBMED, Web of Science, clinicaltrials.gov, and Google Scholar databases was conducted on 31 December 2023, using relevant terms related to EPI, bladder cancer, and NMIBC. The inclusion criteria targeted studies that evaluated patients treated with EPI following the transurethral resection of bladder tumors (TURBT) for NMIBC and compared oncological outcomes such as recurrence and progression with other adjuvant therapies, including Mitomycin C (MMC), Gemcitabine (GEM), and Bacillus Calmette-Guérin (BCG). Additionally, studies investigating the safety profile of EPI administered intravesically at room temperature and under hyperthermia, as well as oncological outcomes associated with hyperthermic intravesical EPI administration, were included. **(3) Results:** Eleven studies reported adverse events after adjuvant intravesical instillations with EPI; the most frequently reported adverse events included cystitis (34%), dysuria, pollakiuria, hematuria, bladder irritation/spasms, fever, nausea and vomiting, and generalized skin rash (2.3%). Nine studies compared EPI to BCG in terms of recurrence and progression rates; BCG instillations showed a lower recurrence rate compared to EPI, with limited or non-significant differences in progression rates. Two studies found no significant differences between EPI and MMC regarding progression and recurrence rates. One study showed statistically significant lower recurrence and progression rates with GEM in high-risk NMIBC patients. Another study found no significant differences between EPI and GEM regarding recurrence and progression. **(4) Conclusions**: EPI exhibits similar oncological performances to Gemcitabine and Mitomycin C currently used for adjuvant therapy in NMIBC. Novel delivery mechanisms such as hyperthermia are interesting newcomers.

## 1. Introduction

Bladder cancer (BC) is the second most frequent urological malignancy affecting 573,000 patients worldwide each year [1]. More than 75% of patients diagnosed with bladder cancer have a cancer confined to the mucosa or the lamina propria [2].

The standard treatment for NMIBC is transurethral resection of the bladder tumors (TURBT) followed by additional adjuvant treatment, which comprises single-shot intravesical chemotherapy dose induction +/− maintenance chemotherapy for up to three years [3,4,5]. For low-risk tumors, single instillations of drugs such as postoperative Mitomycin C (MMC), Epirubicin (EPI), or Gemcitabine (GEM) have been found to be effective in reducing disease recurrences [6]. Another meta-analysis study indicated that combined intravesical chemotherapy therapy after TURB is superior to TURB alone in reducing the recurrence rate of NMIB [7].

For intermediate, high-risk, and very high-risk bladder tumors, the most effective adjuvant therapy is with Bacillus Calmette-Guérin (BCG) [8]. BCG therapy is typically given as a series of six weekly instillations followed by a maintenance regimen for up to 3 years.

In the cases of BCG being ineligible or patients being unresponsive, or in the case of BCG shortage, there are some chemotherapeutical agents available such as EPI, MMC, or GEM for intravesical instillations [9], at least six times weekly, but a fixed regimen has been not established yet. A full-year regimen is nowadays considered the minimum for best efficacy of the drugs (six weekly instillations followed by instillation at 6-week intervals for a year) [10]. However, shared decision-making regarding adjuvant therapy with the patient relies also on various factors, including age [11], stage, grade, and risk stratification of bladder cancer, as well as individual patient characteristics such as sarcopenia [12].

As a treatment for bladder cancer, EPI still has a wide spectrum of use in many countries and geographical areas due to its therapeutic efficacy for NMBIC and lack of alternative approved treatments; on the other hand, in other countries such as the US, it is not approved for intravesical treatment [13]. According to European Association of Urology (EAU) guidelines, EPI is an option in patients unfit for BCG or in the case of BCG shortage [2]. In the US alone, it is estimated that more than 8000 patients are not receiving BCG due to a global shortage [14]. So, as alternative adjuvant therapy after TURBT for bladder tumors, EPI has shown time-effectiveness in reducing recurrences. EPI works by interfering with the DNA of cancer cells, preventing their replication and growth [15]. Given the prevalence of non-muscle invasive bladder cancer (NMIBC) and the importance of effective adjuvant therapies, our study aimed to comprehensively assess the efficacy and toxicity profile of Epirubicin (EPI). Specifically, we sought to investigate the recurrence-free survival (RFS) and progression-free survival (PFS) rates associated with EPI treatment compared to those of Bacillus Calmette-Guérin (BCG), Mitomycin C (MMC), and Gemcitabine (GEM). Additionally, we aimed to evaluate the impact of incorporating hyperthermia alongside EPI treatment, particularly in contrast to hyperthermia combined with MMC. Through this analysis, we aimed to provide clinicians and researchers with a clearer understanding of the comparative effectiveness and safety of EPI, as well as the potential benefits of hyperthermia augmentation in NMIBC management.

## 2. Materials and Methods

A systematic search of the MEDLINE, WebofScience, clinicaltrials.gov, and GoogleScholar databases was performed on 31 December 2023, using any combination of the following terms: Epirubicin (EXP) AND bladder cancer (EXP) OR Epirubicin (EXP) AND non-muscle invasive bladder cancer (EXP). All original articles that fulfilled the inclusion criteria were included. We performed additional cross-checking of the reference lists, and “hand searched” for any additional references.

Studies were considered eligible if they included patients with NMIBC; had a prospective or retrospective design; included at least 10 patients; and assessed the oncological impact of EPI treatment compared with those after BCG, MMC, and GEM or EPI standard treatment alone or using chemohyperthermia. The language of publication was not an exclusion criterion. The primary outcomes were a comparison of recurrence and progression rates between EPI and MMC, GEM, or BCG. The secondary outcome was to evaluate the safety profile of EPI and the impact of using device-assisted intravesical administration of EPI. For each selected study, the following items were recorded: first author’s name, year of publication, country, study design, number of patients, patient’s characteristics, variables included in multivariable analysis, recurrence rate, progression rate, follow-up, and adverse events (AEs), when reported. The study was conducted following the necessary protocols for research. Two investigators, S.C. and M.F. (the first two authors), independently conducted literature searches and extracted data from the full-text articles. In case of any discrepancies, these were resolved through consensus, involving a third investigator, M.D.V. (the corresponding author).

## 3. Results

### 3.1. Adverse Events after Intravesical Instillations with Epirubicin

Eleven studies reported adverse events after adjuvant intravesical instillations with EPI, using a regimen of at least six weekly instillations. They included 1165 patients in total, of which 207 were females. The instillation regimen was not uniform as it has no clear recommendation and varied from 6 installations to 17 instillations [16,17,18]. The most frequently reported adverse events were cystitis (34%), followed by dysuria, pollakiuria, hematuria, bladder irritation/spasms, fever, nausea and vomiting, and generalized skin rash (2.3%) [19]; see Table 1.

### 3.2. EPI versus BCG

Nine studies compared EPI to BCG in terms of recurrence and progression rates. They included 1422 patients, of which 316 were females. Prognostic factors included age, gender, number, tumor stage pTa-pT1, and grade G1–G3 (12–19). The recurrence rate was lower for patients treated with BCG instillations [20,21,22,23,24], and regarding progression, the difference was limited or no difference was noticed [20,21]; see Table 2.

### 3.3. EPI versus MMC

Two studies [25,26] had investigated the effect of EPI compared to that of MMC, and they showed that there is no significant differences between the two drugs (EPI vs. MMC) regarding progression and recurrence; see Table 3.

### 3.4. EPI versus GEM

Two studies [27,28] had investigated the effect of EPI compared to that of GEM. They included 459 patients, of which 135 were female. Zhang et al. [28] (This reference was retracted) has shown a statistical significance of low recurrence and progression in patients with high-risk NMBIC, treated with GEM with anHR of 0.165, 95% CI 0.069–0.397, *p* = 0.000, for recurrence and an HR of 0.160, 95% CI 0.032–0.799, *p* = 0.026 for progression. On the other hand, Wang et al. [27] found no statistical significance regarding recurrence and progression; see Table 4.

### 3.5. Chemohyperthermia with Epirubicin

Chiancone et al. [29] looked at the oncological results of EPI as an adjuvant treatment using hyperthermic intravesical chemotherapy (HIVEC) administration. They included 26 patients, of which 18 were males and 8 were females. Recurrence occurred in two patients (7.69%) from the high-grade group and in one (3.85%) from low grade group, and two patients (7.69%) had progression. They concluded that EPI with HIVEC is a valid option of treatment for high-grade NMBIC with BCG intolerance, and there was no difference in oncological outcomes compared to MMC. Similar results were reported also by Arends et al. [30] when using the Synergo device to administer EPI or MMC into the bladder; see Table 5.

## 4. Discussion

The findings from studies investigating adverse events associated with intravesical instillations of Epirubicin (EPI) highlight several key points. Firstly, a substantial number of patients (1165) were included in 11 studies, indicating a robust data set for analysis. The incidence of adverse events varied, with cystitis being the most frequently reported adverse event (34%), followed by dysuria, pollakiuria, hematuria, and others [25,31]. After gemcitabine, the most reported cases were nausea/vomiting (44.2%) and constipation/diarrhea (23.4%) [32]. This underscores the importance of closely monitoring patients undergoing EPI treatment for non-muscle invasive bladder cancer (NMIBC) for these potential complications.

It is acknowledged that BCG treatment demonstrates superior efficacy in preventing disease progression, according to the data presented in the summary table of the analyzed studies (Table 2). The data were already confirmed by the meta-analysis of You et al. [31].

Regarding adjuvant chemotherapy, no clinically significant difference was observed between EPI, MMC, or GEM. The RFS and PFS rates are somewhat similar. However, a recent meta-analysis showed that among 22 studies adopting induction followed by maintenance intravesical therapy, regarding a lower dose of BCG, EPI was associated with a significantly higher risk of recurrence (odds ratio [OR]: 2.82, 95% CI: 1.54–5.15), but not other intravesical chemotherapies, with no significant differences in the risk of progression between intravesical therapies [32]. Further prospective studies are needed to answer which drug has the best tolerability, safety, and impact on oncological outcomes. Some studies are already recruiting patients to test in vitro the drug with the highest antitumor efficacy [33]. Until then, clinicians should use all available therapies based on shared decision-making with the patient and guideline recommendations. Regarding chemohyperthermia, there seems to be some benefit, but it is not yet quantifiable.

A recent randomized clinical trial (RCT) for MMC indicated that the recurrence-free survival (RFS) rate at 24 months was 61% (95% CI 51–69%) in the chemohyperthermia-treated group and 60% (95% CI 50–68%) in the control group (HR 0.92, 95% CI 0.62–1.37; log-rank *p* = 0.8) [34]. These results should be interpreted considering that only the combat bladder recirculation system (Combat Medical, St. Albans, UK) was used for chemohyperthermia instillations, while many other systems are available for intravesical hyperthermia instillations [35], and their use may lead to different outcomes. Additionally, from all available data, no difference was observed in RFS estimates between patients treated with EPI and those treated with MMC [36]. Concerning a comparison with the standard of care, which is BCG treatment, a recent meta-analysis showed no statistically significant difference between chemohyperthermia and BCG as the adjuvant treatment [37]. However, there are little data on this question, and no solid conclusion can be drawn.

Overall, although EPI remains a viable option for the management of NMIBC, the results highlight the need for personalized treatment approaches based on individual patient characteristics and preferences. Further research is needed to elucidate the optimal use of EPI and potential synergies with other therapeutic modalities, including immunotherapy, to improve outcomes for NMIBC patients.

## 5. Conclusions

Epirubicin has meaningful efficacy in addressing NMIBC; however, its efficacy and indications are limited to selected patients, mainly with an intermediate risk according to EAU guideline stratification and to those unfit for or unresponsive to BCG therapy. Retrospective studies highlight that BCG stands out as more effective than Epirubicin in terms of preventing recurrence. Epirubicin exhibits similar oncological performances to Gemcitabine and Mitomycin C currently used for adjuvant therapy in NMIBC. Novel delivery mechanisms such as hyperthermia are interesting newcomers.

## Figures and Tables

**Table 1:** Reported adverse events after intravesical instillation with Epirubicin.

Study/Year	Country	No pts. m/f	No. Instillations	Adverse Reactions No. Patients (%)
Melekos et al., 1993	Greece	84/15	6–8 (50 mg EPI in 50 mL saline)	Cystitis (34%) and hematuria (15%)
Eto et al., 1994	Japan	98/16	(30 mg EPI/30 mL saline) Twice a week/4 weeks Once monthly/11 months	Micturition pain 6 (10.0%), pollakiuria 9 (15.0%), and hematuria 3 (5.0%)
Ryoji et al., 1994	Japan	97	20 mg in 30 mL physiological saline, 17 times for 1 year: once immediately after TUR, once every 2 weeks for the next 4 months, and then once per month for the following 8 months	9.3% (9/97) of the patients’ pain on urination, pollakiuria, and hematuria
Watanabe et al., 1994	Japan	40/13	20 mg EPI was dissolved in 40 mL physiological saline, 17 instillations, seven times at intervals of 2 weeks. Finally, eight intravesical instillations were performed at 1-month intervals. A total of 17 intravesical injections were given over a period of about 1 year	3 cases (5.7%), and most were symptoms of bladder irritation such as pollakiuria
Ali-El-Dein et al., 1997	Egypt	206/47	8 (1/week) (50 mg EPI/40 mL saline) 1 monthly for 12 months (maintenance)	40 to 56% local side effects (contracted bladder)
Okamura et al., 1998	Japan	110/28	(40 mg/mL in normal saline) Arm A (17 instillations) vs. Arm B (6 instillations)	Miction pain and frequency in 10 (7.2%) patients and gross hematuria in 1 (0.7%)
Melekos et al., 1992	Greece	55/10	6 weeks, 1/monthly	Cystitis: 27.9%pts, hematuria 14%, fever 2.3%, nausea and vomiting 2.3%, generalized skin rash 2.3%
Torelli et al., 2001	Italy	130/39	(80 mg/instillation) started within 20 days after TUR—once monthly for 11 months	Chemical cystitis in 9 patients (6.7%), bacterial cystitis in 2 (1.5%)
Bassi et al., 2002	Italy	26/4	6 80 mg EPI (in 50 mL sterile saline)	Grade of toxicity: G1, G2, G3, G4 Bladder spasms/dysuria 4 (13.7%), 9 (31%), 2 (6.89%)– Hematuria–3 (10.3%)– Fever–1 (3%)–
Mitsumori et al., 2004	Japan	51/18	A, delayed (first instillation 7 days after TURBT) and low-dose (30 mg once every 2 weeks, six times) instillations; B, early (three instillations before 7 days after TURBT) and low-doseinstillations; C, delayed and high-dose (30 mg once weekly 12 times) instillations; D, early and high-doseinstillations	18 patients (26%): irritated bladder 13 pts (18.84%), hematuria 1 pt (1.44%), and bacterial cystitis 4 pts (5.79%)
Kato et al., 2015	Japan	71/17	30 mg of EPI plus 200 mg of Ara-C dissolved in 20 mL of physiological saline weekly for the first year, then every 2 weeks for the second year, once a month for the third year, and once every 3 months during the fourth and fifth years	Severe, reversible cystitis 2 pts (4.5%)

Legend: EPI: Epirubicin; TURBT: transurethral resection of bladder tumor, Ara-C: Cytosin Arabinoside.

**Table 2:** Studies comparing recurrence and progression rates after treatment with Epirubicin and Bacillus Calmete-Guerin.

Study/Year	Country	Design (Period)	No pts. m/f	Age Median (IQR)	Stage	Grade	Variables	Recurrence	Progression	Follow-Up
Duchek et al., 2009	Sweden	Prospective study February 1999–December 2006	256	67	T1	BCG G2 35% (28) 32% (26) EPI G3 91% (72) 92% (74)	drug, size, multifocality, age, Re-TUR, grade, concomitant CIS	34 pts (BCG) vs. 47 pts (EPI&iFN)	No difference regarding the progression	2 years
Marttila et al., 2016	Finland	1997–2008	272	71/70	pTa/pT1/urothelial neoplasm 103/10/2 (90/9/2) 108/6/0 (95/5/0)	BCG G1 75% (65) G2 27% (24) EPI/IFN G1 79% (69) G2 24% (21)	gender, age, no. of recurrences, time to recurrence, multifocality, cytology grade, tumor diameter, perioperative Epirubicin	After 5 years, the recurrence-free estimate of the BCG group was significantly better than that of the EPI/IFN group, 59% versus 38%, respectively	There was no significant difference in the probability of progression or overall survival	BCG/EPI 7.5 years/7.4 years
Tozawa et al., 2001	Japan	March 1990 to February 1999	72	70 years	BCG pTa 13 pT1 37 EPI pTa 7 pT1 57	BCG G1 14 G2 34 G3 2 EPI G1 6 G2 50 G3 8	age, sex, tumor grade, stage, number of recurrences before TURBT	32.0% (16/50) in BCG-treated patients 26.1% (6/23) of patients with chemoimmunotherapy	However, the comparison of Kaplan–Meier curves at the 3-year time point revealed a lower tumor recurrence in the BCG monotherapy group, significant at a level of *p* = 0.026	2 years
Melekos et al., 1996	Greece	Prospective Study	132	BCG/EPI 65.3/67.2	BCG Ta 34 T1 24 EPI Ta 38 T1 23	BCG G1 12 G2 34 G3 12 EPI G1 12 G2 35 G3 14	gender, age, primary tumors, multiple tumors, stage, grade, previous intravesical therapy, concomitant CIS	Free of recurrence 44% for Epirubicin vs. 55% for BCG 10 (16.4%) in the Epirubicin group and 7 (12%) in the BCG	10 (16.4) EPI vs. 7 (12) BCG	2 years
Chi Wai Cheng et al., 2004	China	Between July 1988 and September 1999	36	71.6 years	T1	G3	N.A	16 pts (44.4%)	9 pts (25%)	12 years
Chi Wai Cheng et al., 2005	China	Between October 1991 and September 1999	209	69.9 years	BCG Ta 63 T1 39 EPI Ta 77 T1 29	BCG G1 19 G2 47 G3 33 EPI G1 30 G2 55 G3 20	N.A	59 pts had recurrence with EPI vs. 30 pts with BCG	The 10-year Kaplan–Meier estimate for progression-free survival was 78% in BCG vs. The 10-year Kaplan–Meier estimate for progression-free survival was 74% in EPI	23 months
Iida et al., 2009	Japan	Retrospective study between January 1991 and September 2005	93	73.95 years	EPI T1/G3 69 pts BCG T1/G3 24 pts	G3	sex, age, multifocality, stage, grade, previous intravesical therapy	31 pts (33%)	14 pts—cancer progression	68.7 months
Hemdan et al., 2013	Sweden	Prospective study Between 1999 and 2006	256		BCG T1G2-3 126 pts EPI + IFN T1G2-3 124 pts	G2-3	risk of recurrence, treatment failure, cancer-specific death	5 years BCG vs. Epi + IFN 59% vs. 38%	Free of progression 78% and 77%	6.9 years
Melekos et al., 1993	Greece	Prospective trial	190	Epi 65.8 y BCG 67.1 y	EPI Ta: 42 T1: 25 BCG Ta: 41 T1: 21	EPI G1:31 G2:25 G3:11 BCG G1:27 G2:27 G3:8	gender, age, primary tumors, multiple tumors, stage, grade, previous intravesical therapy, concomitant CIS	EPI 27 (40.3) BCG 20 (32.2)	EPI 6 (9) BCG 4 (6.5)	32.9 months

Legend: BCG: Bacillus Calmete-Guerin, EPI: Epirubicin, IFN: interferon, CIS: carcinoma in situ.

**Table 3:** Studies comparing recurrence and progression rates after adjuvant treatment with Epirubicin or MMC in patients with non-muscle invasive bladder cancer.

Study/Year	Country	Design (Period)	No pts. m/f	Age Median (IQR)	Stage	Grade	Variables	Recurrence	Progression	Follow -Up
Bono et al., 1996	Italy	October 1986–April 1989	108	65.5 years	Study (30,864) (MMC) Ta in 82 patients (76%) T1 in 26 (24%) Study (30,869) (EPI) Ta in 35 patients (87.5%) T1 in 5 patients (12.5%)	Study (30,864) (MMC) G1 in 33 cases (30.6%), G2 in 67 cases (62.0%), and G3 in 8 cases (7.4%). Study (30,869) (EPI) G1 in 15 cases (37.5%), G2 in 22 cases (55.0%), and G3 in 3 cases (7.5%)	<85 years, good general health, multiple primary or recurrent Ta-T1	Treated with MMC 19 pts–19.79%	progression in 20% of patients	N.A
Calais da Silva et al., 1992	Portugal	N.A	46/14	68 years	EPI Ta6 patients T1 23 patients MMC Ta 1 patient T1 17 patients	EPI G1—11 patients G2—14 patients G3—7 patients MMC G1—10 patients G2—16 patients G3—2 patients	Single/multiple tumor Primary-recurrent	EPI Primary Ta 6 patients with 1 recurrence; primary T1-23 patients with 8 recurrences, and recurrent T3 patients with 3 recurrences. MMC Ta 1 patient with no recurrence; primary T1 17 patients with 5 recurrences; recurrent Ta 2 patients with no recurrences, and T 8 patients with 3 recurrences.	N.A	17.7 months

Legend: MMC: Mytomicin C; EPI: Epirubicin; N.A: not available.

**Table 4:** Studies comparing recurrence and progression rates after adjuvant treatment with Epirubicin or Gemcitabine in patients with non-muscle invasive bladder cancer.

Study/Year	Country	Design (Period)	No pts. m/f	Age Median	Stage	Grade	Variables	Recurrence	Progression	Follow -Up
Wang et al., 2019	China	January 1996 to July 2018	91/33 f	N.A	N.A	GEM Low 42 (57.53%) High 31 (42.47%) EPI Low 19 (51.35%) High 18 (48.65%)	gender, age, multifocality, size, grade, risk, re-TURBT	Gemcitabine intravesical chemotherapy group was significantly related to a lower rate of recurrence in GEM (HR = 0.165, 95% CI 0.069–0.397, *p* = 0.000)	lower rate of progression with GEM (HR = 0.160, 95% CI 0.032–0.799, *p* = 0.026)	GEM 34.8 months EPI 35.9 months
Zhang et al., 2021	China	Retrospective study from October 2015 to October 2019	233/102 f	62 years	Ta A29 B30 C36 T1 A38 B51 C38	Low Grade A34 B40 C48 High Grade A33 B41 C26	gender, age, size, number of tumors, stage, grade	*p* = 1.00—no statistical significance	*p* = 0.69—no statistical significance	

Legend: GEM: Gemcitabine, EPI: Epirubicin, TURBT: transurethral resection of bladder tumors, N.A: not available.

**Table 5:** Studies comparing recurrence and progression rates after adjuvant treatment with Epirubicin or MMC using hyperthermia in patients with non-muscle invasive bladder cancer.

Study/Year	Country	Design (Period)	No. Patients Male/ Female	Chemohyperthermia	Characteristics	Age Years Mean/SD	Stage/Grade	Variables	Recurrence	Progression	Follow -Up
Chiancone et al., 2020	Italy	Retrospective March 2017–February 2020	98/33 (33.7%)	HIVEC 72 pts. MMC vs. 26 pts. EPI	BCG failure or intolerance patients with high-risk NMIBC	67.54 ± 7.96 vs. 64.35 ± 8.56	Ta G3 15 (79.17%) vs. 11 (57.69%) T1G3 57 (20.83%) vs. 15 (42.31%)	Age, gender, smoking status, BMI, diabetes, number of tumors, tumor size, recurrence rate, pathologic state, concomitant CIS, tumor on RE-TURB, previously treated with MMC, BCG failure group	High-grade 14/72 (19.44%) MMC vs. 2/26 (7.69%) EPI Low-grade 3/72 (4.17%) MMC vs. 1/26 (3.85%) EPI	MMC 4/72 (5.56%) vs. EPI 2/26 (7.69%)	10.5 vs. 14 months
Arends et al., 2014	The Netherlands	Prospective maintain database 2002–2013	160/36 (22.5%)	Synergo SB-TS 101 system 20 EPI 140 MMC	NMIBC refractory to regular intravesical treatment	65 (range 34 to 87)	pT1 75 (46.9%), pTa 85 (53.1%), high-grade 104 (65.0%), low-grade 56 (35.0%)	Age, gender, CIS history, No. preCHT TURBTs, PreCHT T1 on histology, PreCHT highly recurrent NMIBC, PreCHT grade	1-year RFS 64% EPi vs. 59% MMC 2-year RFS 55% EPI vs. 46% MMC, (*p* = 0.303)	N.A	75.6 months

Legend: HIVEC: hyperthermic intravesical chemotherapy; MMC: Mitomycin C; EPI: Epirubicin; SD: standard deviation; NMIBC: non-muscle invasive bladder cancer, TURBT: transurethral resection of the bladder tumors; CIS: carcinoma in situ; CHT: chemohyperthermia; BCG:Bacillus Calmette-Guerin; BMI: body mass index; N.A: not available.

## Data Availability

Not applicable.
